# Comparison of Health Care Spending by Age in 8 High-Income Countries

**DOI:** 10.1001/jamanetworkopen.2020.14688

**Published:** 2020-08-06

**Authors:** Irene Papanicolas, Alberto Marino, Luca Lorenzoni, Ashish Jha

**Affiliations:** 1 Department of Health Policy, London School of Economics, London, United Kingdom; 2 Department of Health Policy and Management, Harvard T.H. Chan School of Public Health, Boston, Massachusetts; 3 Health Division, Organisation for Economic Co-operation and Development, Paris, France; 4 Harvard Global Health Institute, Cambridge, Massachusetts

## Abstract

This cross-sectional study compares health care spending by age in the US with spending in 8 other high-income countries.

## Introduction

The United States spends more on health care than any other country.^1^ Unlike many other high-income countries, which have largely uniform financing schemes for health care, the US has different financing schemes for different populations. The degree to which this fragmentation in US financing explains higher spending is not clear. Some policy makers believe that expanding the Medicare model, which has a financing system that more closely resembles that of other high-income countries (ie, it is government run and tax financed), could reduce spending substantially. To examine whether this policy has potential, this cross-sectional study compared nominal and relative spending in the US, by 5-year age groupings, with that of other high-income countries that have more homogenous financing systems. This comparison allows us to better understand spending differentials between the US and other countries for people aged 65 years or older, as well as for other age groups.

## Methods

This cross-sectional study was granted exemption from institutional review board approval and informed consent by the London School of Economics because the data used are publicly available and cannot be linked back, directly or indirectly, to any individuals. This article followed the relevant portions of the Strengthening the Reporting of Observational Studies in Epidemiology (STROBE) reporting guidelines. We used data from the Organisation for Economic Co-operation and Development^2^ to examine variations in total current health care spending per capita, by age cohort, for the US and 7 other high-income countries (ie, Australia, Canada, Germany, Japan, the Netherlands, Switzerland, and the United Kingdom) in 2015. These data were derived from national sources and the “Health Expenditures by Diseases and Conditions” report.^3^ For the US, per capita health spending by age cohort was derived from 2013 Institute for Health Metrics and Evaluation expenditure. Data on the US 2015 population structure were obtained from the United Nations “World Population Prospects: The 2017 Revision.”^4^ Expenditure data were translated into US dollar equivalents using 2015 actual individual consumption purchasing power parities from the Organisation for Economic Co-operation and Development.

## Results

For all 7 comparator countries, the mean (SD) per capita spending in health care was $4924 ($937). In the US, per capita health care spending was $9524, or 1.9-fold higher than the mean for the 7 comparator countries. The Figure illustrates US and comparator countries’ health care expenditures per capita by age cohort. The absolute difference between US spending and that of the other countries for ages 0 to 4 years was $3899, and that difference decreased at approximately age 5 years, after which it slowly increased. The difference increased faster after age 65 years, peaking at $18 645 for ages 80 to 84 years.

**Figure.**
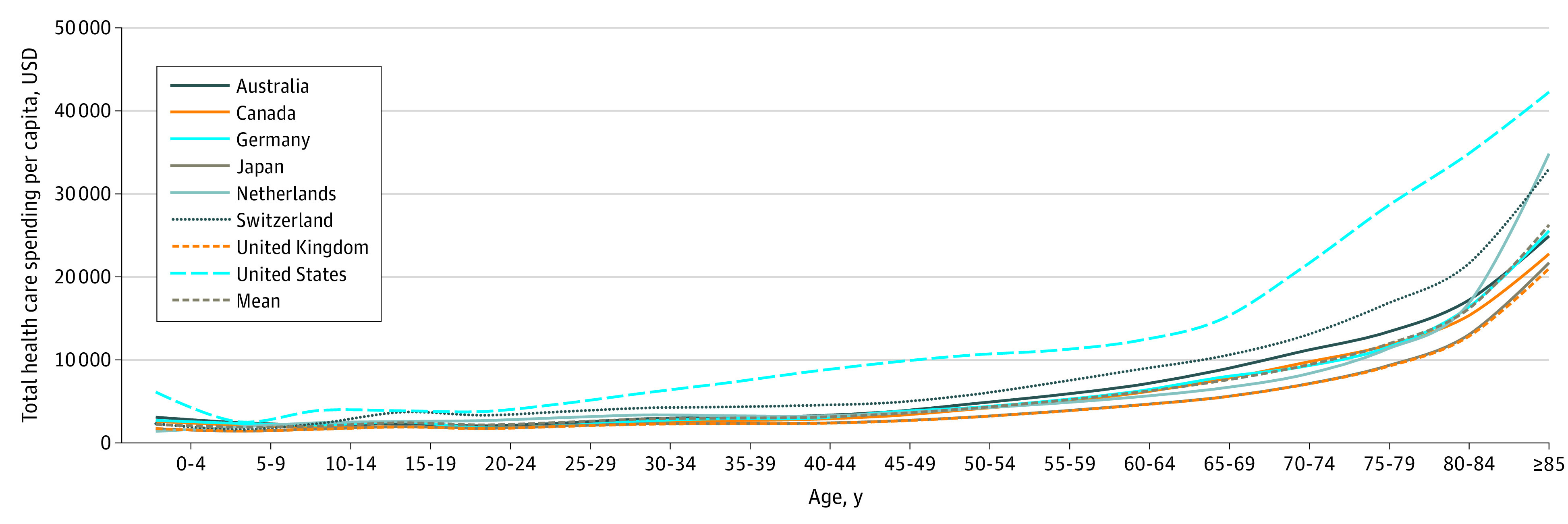
Per Capita Health Care Spending by Age Group in 8 High-Income Countries in 2015 Spending is purchasing power parity–adjusted. The mean includes all countries except the US. USD indicates US dollars.

The Table illustrates differences in US spending relative to the comparator mean for 3 broader groups: youths (ages 0 to 19 years), adults (ages 20 to 64 years), and older adults (65 years and older), as well as the entire population. The gap in per capita health care spending in the US vs the mean (SD) of comparator nations was highest for the adult age group, at $8161 vs $3603 ($753), a difference of 2.3-fold the mean for the 7 comparator nations. The gap in per capita health care spending between the US and the mean (SD) of comparator nations decreased for individuals aged 65 years and older ($24 665 vs $12 309 [$2213]; difference relative to mean: 2.0) and for those aged 0 to 19 years ($4097 vs $2166 [$867]; difference relative to the mean: 1.9).

**Table.** Per Capita Health Care Spending by Age Group in the US and Comparator Countries in 2015

Country	Per capita health expenditure, USD				Expenditure relative to the mean			
	Age group, y				Age group, y			
	0-19	20-64	≥65	All	0-19	20-64	≥65	All
United States	4097	8161	24 655	9524	1.9	2.3	2.0	1.9
Australia^a^	2525	3771	13 316	4888	1.2	1.0	1.1	1.0
Canada	2147	3366	11 773	4457	1.0	0.9	1.0	0.9
Germany	2448	3630	12 442	5 277	1.1	1.0	1.0	1.1
The Netherlands	2115	3763	12 285	4916	1.0	1.0	1.0	1.0
Japan	1711	2822	9972	4486	0.8	0.8	0.8	0.9
Switzerland	2530	5166	16 788	6730	1.2	1.4	1.4	1.4
United Kingdom	1686	2705	9584	3714	0.8	0.8	0.8	0.8
Mean (SD)^b^	2166 (334)	3603 (753)	12 309 (2213)	4924 (867)	1.0	1.0	1.0	1.0

^a^ Total current health spending for Australia was increased by 11.5% from the Organisation for Economic Co-operation and Development base figure to account for residential aged care expenditure, which is classified under welfare (social) expenditure in Australia instead of health expenditure as in other Organisation for Economic Co-operation and Development countries.

^b^ The mean values represent the mean of comparator countries (excluding the US). Values are in purchasing power parity–adjusted 2015 US dollars.

## Discussion

This cross-sectional study found that the US spent a mean of 1.9-fold more on health care per capita compared with the mean of 7 high-income countries. The ratio of spending in the US to that in comparator countries was lower for people aged 65 years and older (2.0-fold the mean) than for those aged 20 to 64 years (2.3-fold). However, the Medicare-eligible population in the US still spent 100% more per capita on health care than older adults spent in the 7 comparator countries. In addition, the narrowing in the spending gap for individuals aged 65 and older was driven by substantial increases in spending among those aged 85 years and older in the comparator nations, not by a reduction in spending in the US older population. Moreover, in absolute dollar amounts, not ratios, this spending gap actually increased to be the largest among individuals 65 years and older, with the typical person in the US spending nearly $18 600 more at approximately age 80 years than the typical person in these other high-income countries. Greater spending on health care in the US is likely associated with various factors, including health status,^1,5^ health care prices,^6^ and the breadth of services covered.

This study has some limitations, including differences in local data collection and accounting methods, as well as variability across national benefit packages, particularly long-term care. These differences may influence comparability. Additionally, the data presented are purely descriptive and do not explain which factors contribute to per capita health care spending at different ages.

Our findings suggest that despite appearing similar in structure to the health care systems of other high-income countries, the US health care system for individuals aged 65 years and older is comparably more costly. These findings suggest that moving to a Medicare-for-all model may not substantially reduce US health care spending relative to that of other high-income countries. Different approaches are likely needed if the US is to adopt a system that achieves this aim.
